# Synaptic Neurotransmission Depression in Ventral Tegmental Dopamine Neurons and Cannabinoid-Associated Addictive Learning

**DOI:** 10.1371/journal.pone.0015634

**Published:** 2010-12-20

**Authors:** Zhiqiang Liu, Jing Han, Lintao Jia, Jean-Christian Maillet, Guang Bai, Lin Xu, Zhengping Jia, Qiaohua Zheng, Wandong Zhang, Robert Monette, Zul Merali, Zhou Zhu, Wei Wang, Wei Ren, Xia Zhang

**Affiliations:** 1 College of Life Sciences, Shaanxi Normal University, Xian, People's Republic of China; 2 Institute of Mental Health Research and Departments of Psychiatry and Cellular & Molecular Medicine, University of Ottawa, Ottawa, Canada; 3 Department of Neural and Pain Sciences, Dental School, Program in Neuroscience, University of Maryland, Baltimore, Maryland, United States of America; 4 Key Laboratory of Animal Models and Human Disease Mechanisms, Kunming Institute of Zoology, Chinese Academy of Science, Kunming, People's Republic of China; 5 Neurosciences and Mental Health, The Hospital for Sick Children, Toronto, Ontario, Canada; 6 Neurobiology Program, Institute for Biological Sciences, National Research Council of Canada, Ottawa, Canada; 7 Department of Neurology, Tongji Hospital, Tongji Medical College, Huazhong University of Science and Technology, Wuhan, People's Republic of China; Medical College of Georgia, United States of America

## Abstract

Drug addiction is an association of compulsive drug use with long-term associative learning/memory. Multiple forms of learning/memory are primarily subserved by activity- or experience-dependent synaptic long-term potentiation (LTP) and long-term depression (LTD). Recent studies suggest LTP expression in locally activated glutamate synapses onto dopamine neurons (local Glu-DA synapses) of the midbrain ventral tegmental area (VTA) following a single or chronic exposure to many drugs of abuse, whereas a single exposure to cannabinoid did not significantly affect synaptic plasticity at these synapses. It is unknown whether chronic exposure of cannabis (marijuana or cannabinoids), the most commonly used illicit drug worldwide, induce LTP or LTD at these synapses. More importantly, whether such alterations in VTA synaptic plasticity causatively contribute to drug addictive behavior has not previously been addressed. Here we show in rats that chronic cannabinoid exposure activates VTA cannabinoid CB1 receptors to induce transient neurotransmission depression at VTA local Glu-DA synapses through activation of NMDA receptors and subsequent endocytosis of AMPA receptor GluR2 subunits. A GluR2-derived peptide blocks cannabinoid-induced VTA synaptic depression and conditioned place preference, i.e., learning to associate drug exposure with environmental cues. These data not only provide the first evidence, to our knowledge, that NMDA receptor-dependent synaptic depression at VTA dopamine circuitry requires GluR2 endocytosis, but also suggest an essential contribution of such synaptic depression to cannabinoid-associated addictive learning, in addition to pointing to novel pharmacological strategies for the treatment of cannabis addiction.

## Introduction

Cannabis (marijuana or cannabinoids) is the most commonly used illicit drug worldwide [1] and the lifetime prevalence of cannabis addiction is the highest of all illicit drugs in the United States [2]. However, there is no effective treatment for cannabis addiction in humans, largely due to our poor understanding of its underlying mechanism. The current view of drug addiction emphasizes an association of compulsive drug use with molecular and cellular mechanisms underlying long-term associative learning and memory [3], [4]. Ample evidence supports activity- or experience-dependent long-term changes of synaptic strength, i.e., long-term potentiation (LTP) and long-term depression (LTD), as the primary cellular mechanisms underlying multiple forms of learning and memory [5]. Therefore, it would be of interest to examine the relationship of long-term changes of synaptic strength with drug addiction in the brain circuitry critically involved in drug addiction, such as the midbrain ventral tegmental area (VTA) where most drugs of abuse, including cannabis, prominently increase the activity of its dopamine neurons and thereby lead to drug rewarding response [6], [7].

Stimulation of local excitatory afferents in the VTA activates both postsynaptic AMPA receptor (AMPAR) and NMDA receptor (NMDAR) in VTA dopamine neurons [8], [9]. AMPAR consists of GluR1–GluR4 subunits [10], whereas NMDAR is heteromeric complex of NR1 subunit and at least one type of four NR2 subunits (NR2A–NR2D) [11]. Recent studies showed an induction of NMDAR-dependent LTP at the locally activated glutamate synapses onto VTA dopamine neurons (local Glu-DA synapses) following a single exposure of animals to nicotine, cocaine, amphetamine, morphine and ethanol [12], [13] and facilitated LTP induction at these synapses following chronic exposure to cocaine [14] or when pairing of nicotine application with depolarizations [15]. These reports suggest that LTP expression in VTA local Glu-DA synapses following *in vivo* exposure of drugs of abuse may play an important role in the development of drug addiction [12]–[15]. A more recent study further demonstrated that a single exposure to cocaine potentiated both VTA local Glu-DA synapses and pedunculopontine nucleus-activated glutamate synapses onto VTA dopamine neurons (PPN Glu-DA synapses) [16]. This potentiation occurred through insertion of the higher conducting GluR2-lacking AMPAR into both synaptic pathways, which subsequently permitted expression of metabotropic glutamate receptor (mGluR)-dependent LTD through reinsertion of the lower conducting GluR2-containing AMPAR at these synapses [16]. It is interesting to note that a single exposure of the major psychoactive ingredient of marijuana, Δ9-tetrahydrocannabinol (THC), induced GluR2-lacking AMPAR insertion into the PPN Glu-DA synapses without significant effects on the local Glu-DA synapses, thus permitting subsequent expression of mGluR-dependent LTD through GluR2-containing AMPAR reinsertion at the PPN Glu-DA synapses [16].

In summary, it is known that both a single and chronic exposure to cocaine likely induces LTP at VTA local Glu-DA synapses [12]–[14], which are generally believed to encode the powerful glutamate afferent neurotransmission originating from the cerebral cortex, especially the prefrontal cortex [17]. It is unknown, however, whether chronic exposure of cannabinoids induces LTP or LTD in VTA local Glu-DA synapses. More importantly, whether such alterations in VTA synaptic plasticity causatively contribute to drug addictive behavior has not previously been addressed. To examine these issues, in the present study we performed field potential recording of the EPSP (fEPSP) from the VTA without any receptor antagonists for two reasons. First, field potential recording of the EPSP, but not whole cell recording of the excitatory postsynaptic current (EPSC), is able to provide the essential information regarding *overall* change in excitatory afferent synapses onto *a population of* various types of VTA neurons. Second, the absence of any receptor antagonists during recording would allow us to evaluate the overall influence of all receptors on excitatory afferent synapses onto VTA neurons. To overcome the potential problems of field potential recording (f.g., not able to differentiate the type of cells expressing LTD/LTP), we further conducted whole cell recordings of the EPSC from individual dopamine and GABA neurons in the VTA. Finally, we explored the mechanism underlying cannabinoid-induced changes in synaptic strength in the VTA and its relation with cannabis-associated addictive behavior by employing a combination of field potential recordings and molecular, biochemical, immunohistochemical and behavioral strategies.

## Results

### Cannabinoids Facilitate LTD Induction in the VTA via CB1 and NMDA Receptors

**Figure 1:**
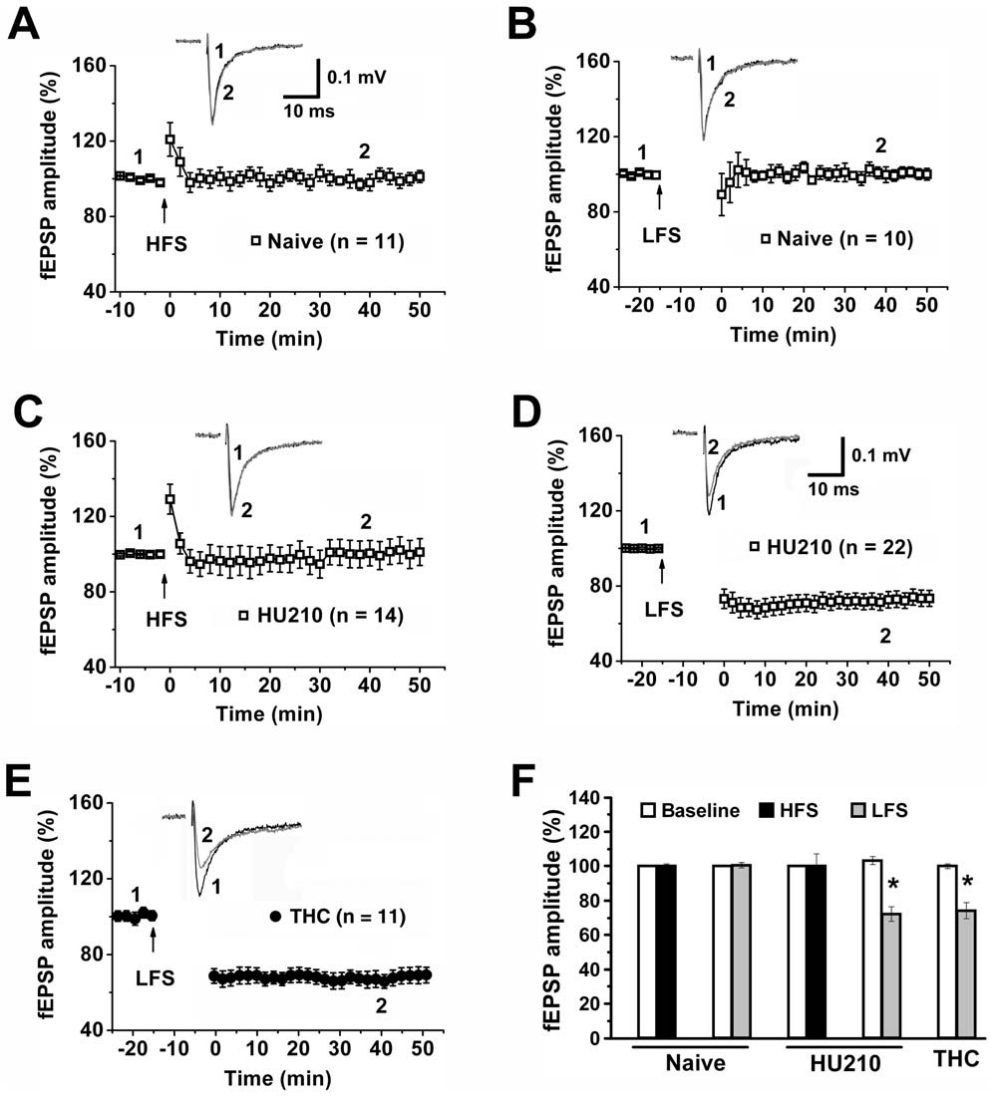
Cannabinoids facilitate LTD induction in the VTA. (A) HFS failed to induce LTP in naïve rat VTA. (B) LFS failed to induce LTD in naïve rat VTA. (C) HFS failed to induce LTP from the VTA of HU210-treated rats. (D) LFS induced LTD from the VTA of chronic HU210-treated rats. (E) LFS induced LTD from the VTA of chronic THC-treated rats. (F) Summary of LTD induction under various conditions. * *p*<0.01 versus baseline.

To determine the impact of cannabinoid on VTA synaptic plasticity, we recorded the field EPSPs (fEPSPs) in midbrain slices prepared from naïve rats and rats killed 24 h after chronic injection (once daily injections for 5 days) of the cannabinoid HU210 (100 µg/kg, i.p.) or vehicle. It has been shown that high-frequency stimulation (HFS) and low-frequency stimulation (LFS) of VTA local excitatory afferents induced LTP and LTD, respectively, in the local Glu-DA synapses of naïve rats with the presence of picrotoxin in the bath solution [8], [17]. We observed here that without picrotoxin in the bath solution, neither HFS (100 Hz) nor LFS (1 Hz, 15 min) induced LTP or LTD in the VTA of naïve rats (Figure 1A,B,F) or vehicle-treated rats (100.3±6%, 100.1±4%, *p*>0.05 versus control, *n* = 11). In contrast, LFS induced LTD (72.3±4.3%, *p*<0.01 versus control, *n* = 22), but HFS still failed to induce LTP (100.2±7%, *p*>0.05 versus control, *n* = 14), from HU210-treated rats (Figure 1C,D,F). To explore whether other cannabinoids can also facilitate LTD induction in the VTA, we examined THC (5 mg/kg, i.p.), the major psychoactive ingredient of marijuana. Similar to HU210 injection, chronic THC injection also facilitated LFS-induced LTD in the VTA (74.1±4.6%, *p*<0.01 versus control, *n* = 11) (Figure 1E,F).

**Figure 2:**
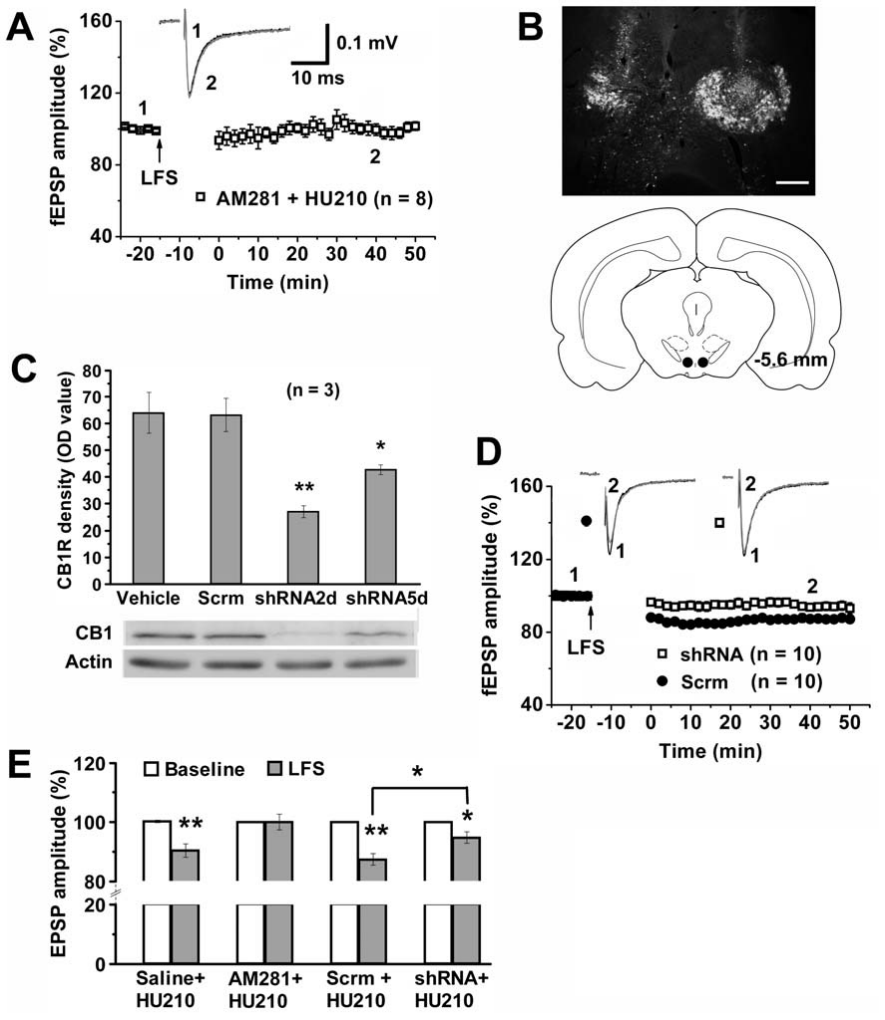
VTA CB1R mediates cannabinoid-facilitated LTD induction in the VTA. (A) Systemic pretreatment with the selective CB1R antagonist AM281 prior to daily HU210 injection blocked LFS-induced LTD in the VTA. (B) Localization of bilaterally microfusioned adenoviral vectors containing GFP into the VTA. Scale bar: 150 µm. (C) Graph (top) and immunoblotting (bottom photos) show that CB1R shRNA (shRNA), but not its scrambled sequence (Scrm), reduced CB1R expression in the VTA 2 and 5 days after intra-VTA injection. One-way ANOVA: *F*_3,8_ = 11.949, *p*<0.01. * *p*<0.05, ** *p*<0.01 versus vehicle (LSD post hoc test). (D) Relative to scrambled shRNA (Scrm), CB1R shRNA (shRNA) infusion into the VTA inhibited LFS-induced LTD in HU210-treated rats. (E) Summary of LTD induction following treatment with saline, AM281, scrambled shRNA (Scrm) or CB1R shRNA (shRNA). One-way ANOVA: *F*_7,70_ = 14.853, *p*<0.001. * *p*<0.01, ** *p*<0.001 (LSD post hoc test) versus baseline or between Scrm+HU210 and siRNA+HU210.

Because both HU210 and THC are able to activate both cannabinoid CB1 receptor (CB1R) and CB2 receptor, we further determined the role of CB1R in cannabinoid-facilitated LTD induction in the VTA. Daily pretreatment of rats with the selective CB1R antagonist AM281 at the dose (3 mg/kg, i.p.) that blocked cannabinoid action shown in our recent study [18], but not vehicle, 20 min before daily HU210 injection prevented HU210-facilitated LTD induction (100±2.6%, *p*>0.05 versus control, *n* = 8) in the VTA (Figure 2A,E). We then examined LTD induction after a selective knocking down of CB1R expression in the VTA using CB1R shRNA expressed by adenoviral vectors. After a bilateral injection of the vectors into the major part of the VTA (Figure 2B), the CB1R shRNA, but not its scrambled sequence, significantly suppressed CB1R expression in the VTA (Figure 2C) and LFS-induced LTD in HU210-treated rats (Figure 2D,E).

**Figure 3:**
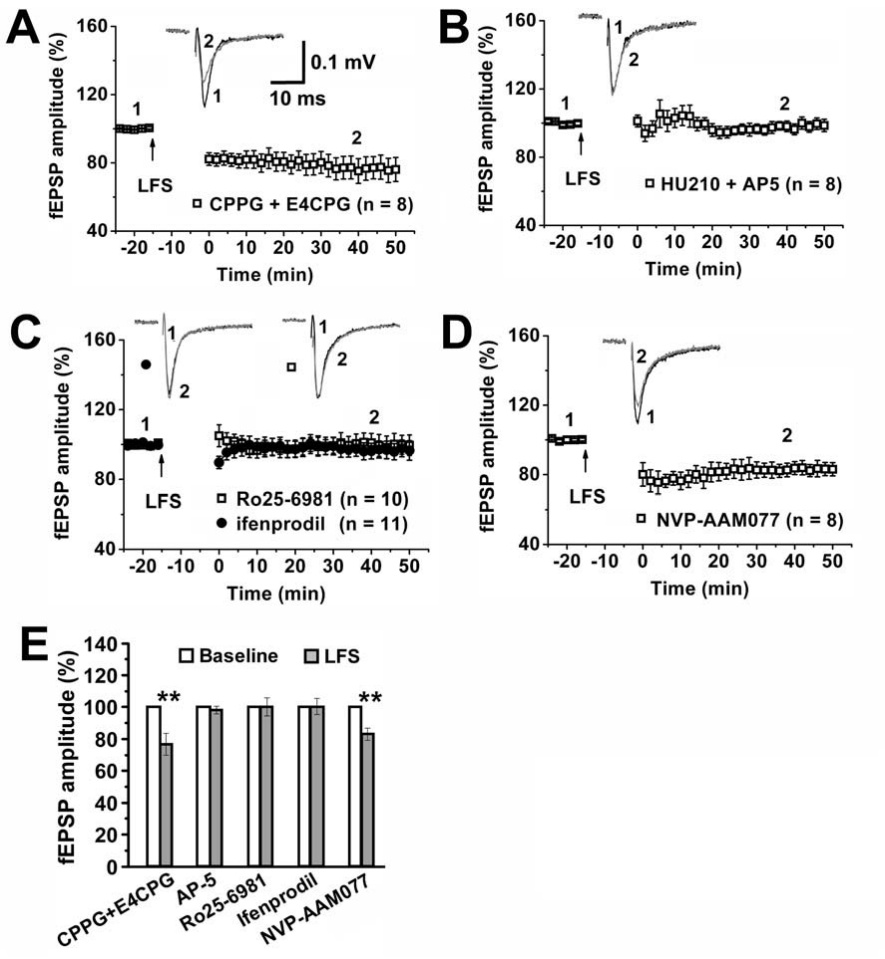
NMDAR mediates cannabinoid-facilitated LTD induction in the VTA. (A) CPPG and 4CPG together failed to affect LFS-induced LTD in chronic HU210-treated rats. (B) AP-5 blocked LFS-induced VTA LTD in chronic HU210-treated rats. (C) Ro25-6981 and ifenprodil blocked LFS-induced VTA LTD in chronic HU210-treated rats. (D) NVP-AAM077 failed to affect LFS-induced LTD in chronic HU210-treated rats. (E) Summary of LTD induction and blockade. ** *p*<0.01 versus baseline.

LTD induction can be divided into NMDAR-dependent and NMDAR-independent LTD [5], [19]. We first examined mGluR, because cannabinoids induce LTD in the nucleus accumbens via mGluR [20]. Bath application of vehicle or both the selective I/II mGluR antagonist (*RS*)-α-ethyl-4-carboxyphenylglycine (CPPG, 10 µM) and II/III mGluR antagonist (*RS*)-α-cyclopropyl-4-phosphonophenyl-glycine (4CPG, 10 µM) at concentrations that block mGluR activity [21], [22] failed to affect LTD induction in HU210-treated rats (Figure 3A, 3E). In contrast, the selective NMDAR antagonist AP-5 (50 µM) blocked LTD induction in HU210-treated rats (98.1±2.3%, *p*>0.05 versus control, *n* = 8) (Figure 3B,E). NMDARs in adult brain are heteromeric complexes of NR1 and NR2A or NR2B subunit. NVP-AAM077 (0.4 µM) has been shown to completely block NR2A-containing NMDAR and attenuate NR2B-containing NMDAR [23], [24]. Ro25-6981 (1 µM) and ifenprodil (10 µM) have been shown to selectively block NR2B-containing NMDAR [25], [26]. We found that Ro25-6981 (1 µM) or ifenprodil (10 µM), but not NVP-AAM077 (0.5 µM), blocked LFS-induced LTD (Figure 3C,D,E), suggesting an involvement of NR2B-containing NMDAR in cannabinoid-facilitated LTD induction in the VTA.

### Cannabinoid Facilitates LTD Expression in VTA Dopamine Neurons via AMPAR

**Figure 4:**
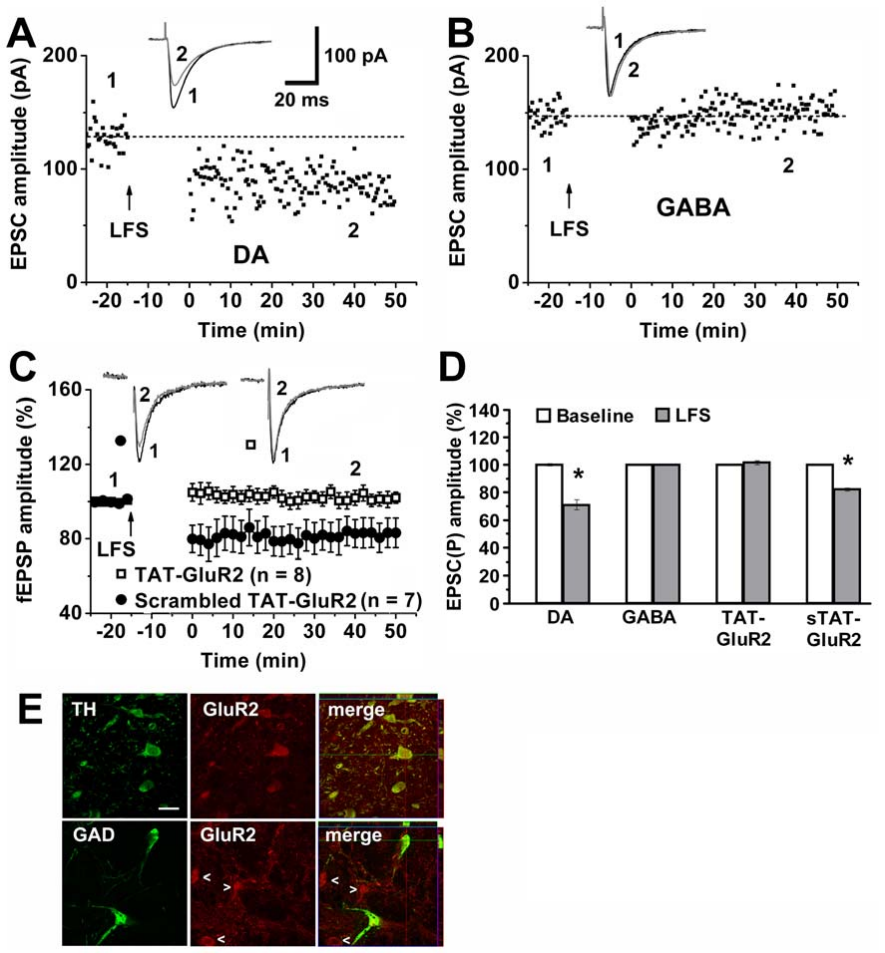
Dopamine neurons express cannabinoid-facilitated LTD in the VTA. (A) LFS induced a LTD from a VTA dopamine neuron (DA) in chronic HU210-treated rats. (B) LFS did not induce LTD from a VTA GABA neuron (GABA) in chronic HU210-treated rats. (C) TAT-GluR2 peptide, but not scrambled TAT-GluR2 peptide, prevented VTA LTD induction in HU210-treated rats. (D) Summary of LTD induction and blockade under various conditions. * *p*<0.01 versus baseline. (E) Confocal microscopic images show that GluR2-immunoreactive neurons (red) were doubly stained (yellow) with TH (green), but not with GAD65/67 (green). Scale bar: 25 µm.

Because local VTA glutamatergic afferents directly act on both dopamine and GABA neurons, we employed whole cell recordings to locate the VTA neurons for the expression of cannabinoid-facilitated LTD. Dopamine and GABA cells were identified by the presence and absence of a large (>50 pA) *I*_h_ current, respectively [9]. Although a small number of VTA none-dopamine neurons show a large *I*_h_ current [27], VTA GABAergic neurons do not show a large *I*_h_ current. LFS to midbrain slices from rats receiving chronic HU210 injection induced LTD in all putative dopamine neurons examined (74.6±4.6%, *p*<0.01 versus control, *n* = 6) (Figure 4A,D), but not in any one of all GABA neurons examined (100.1±0.3%, *p*>0.05 versus control, *n* = 8) (Figure 4B,D). Cannabinoid-facilitated LTD in the VTA may therefore be expressed in VTA dopamine neurons but not GABA neurons.

The last step of LTD expression involves facilitated endocytosis of AMPAR GluR2 subunits [5], [28]–[30], which can be selectively blocked by a GluR2-derived peptide (^869^YKEGYNVYG^877^) [28]–[30]. After rendering the peptide with cell-permeable by fusing it to the cell-membrane transduction domain of the TAT protein (YGRKKRRQRRR), the resulting TAT-GluR2 peptide is able to disrupt both GluR2 endocytosis and LTD induction, without significant effects on LTP induction [26], [29]. The pivotal role of GluR2 endocytosis in cannabinoid-facilitated LTD expression in the VTA is supported by our findings that bath application of TAT-GluR2, but not its scrambled sequence, blocked LTD expression in HU210-treated rats (Figure 4C,D). GluR2 endocytosis likely occurs in the postsynaptic membrane of VTA dopamine neurons, instead of GABA neurons, because GluR2-positive neurons were doubly stained with the dopamine-synthesizing enzyme tyrosine hydroxylase (TH), but not with the GABA-synthesizing enzyme glutamic acid decarboxylase 65/67 (GAD65/67) (Figure 4E). These results further support the idea that the expression of cannabinoid-facilitated LTD in the VTA occurs in dopamine neurons, but not in GABA neurons.

### Cannabinoid Transiently Suppresses Synaptic Neurotransmission in VTA Dopamine Neurons

**Figure 5:**
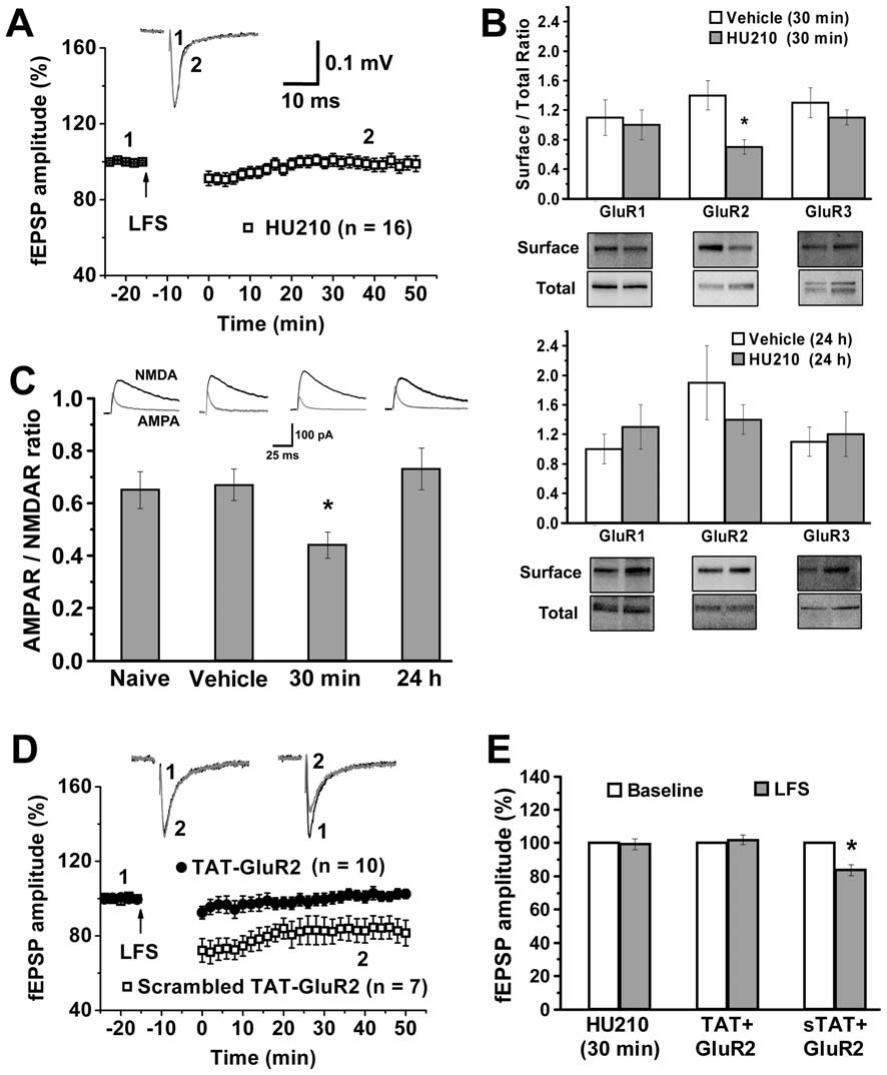
Cannabinoid likely induces *in vivo* LTD in VTA dopamine neurons. (A) LFS did not induce LTD 30 min after the fifth HU210 injection. (B) A selective decrease of surface/whole cell ratio of GluR2 occurred 30 min, but not 24 h, after the fifth HU210 injection, relative to vehicle treatment (n = 3). * *p*<0.05 versus vehicle. (C) The top panel shows sample EPSCs in neurons from naive animals, animals killed 30 min after vehicle injection or animals killed 30 min or 24 h after the fifth daily HU210 injection; the graph shows peak AMPAR- and NMDAR-mediated EPSCs expressed as a ratio. One-way ANOVA: *F*_3,32_ = 4.471, *p*<0.05. * *p*<0.05 versus naïve, vehicle or 24 h. (D) Pretreatment with TAT-GluR2, but not scrambled TAT-GluR2, disrupted LFS-induced LTD in VTA slices prepared 1 day after the fifth HU210 injection. (E) Bar graphs summarize data shown in A and D. * *p*<0.05 versus baseline. HU210 (30 min), midbrain slices were prepared 30 min after the final HU210 injection; sTAT-GluR2, scrambled TAT-GluR2.

While LFS induced VTA LTD at 24 h after chronic injection of HU210 but not vehicle as shown above, LFS failed to induce LTD at 30 min after either the fifth HU210 injection (99.2±3.4%, *p*>0.05 versus baseline, *n* = 16) (Figure 5A,E) or the fifth vehicle injection (101.3±2.9%, *p*>0.05 versus baseline, *n* = 7). These results indicate the possible occlusion of LFS-induced LTD *in vitro* at 30 min, but not at 24 h, by a preceding HU210-induced synaptic depression *in vivo*. This working hypothesis is supported by two additional experiments. First, VTA cell surface levels of GluR2, but not GluR1 or GluR3, significantly reduced 30 min, but not 24 h, after the fifth HU210 injection (*n* = 3) (Figure 5B). Second, the AMPAR/NMDAR ratio of glutamatergic synaptic currents in VTA dopamine neurons was significantly smaller at 30 min (0.44±0.05; *n* = 10) than that at 24 h (0.73±0.08; *n* = 8) after the fifth HU210 injection, that in naïve rats (0.65±0.07; *n* = 10) and that in vehicle-treated rats (0.67±0.06; *n* = 8) while the latter three were not significantly different from each other (Figure 5C).

Our data together indicate that cannabinoid injection induces *in vivo* synaptic depression in VTA dopamine neurons that lasts for less than 24 h. To further test this hypothesis, we applied TAT-GluR2 peptide to block HU210-induced LTD *in vivo*, and then examined whether this blockade would result in prevention of HU210-facilitated VTA LTD induction *in vitro* 24 h after the fifth HU210 injection. Given that TAT-GluR2 peptide reaches peak levels in the rodent brain at 100 min and is washed out of brain within 8 h after a systemic injection [29], we injected TAT-GluR2 (1.5 µmol/kg, i.p.) 2 h prior to each daily HU210 injection. LFS induced VTA LTD in rats receiving scrambled TAT-GluR2 peptide (83.7±6.3%, *p*<0.05 versus control, *n* = 7), but not in rats receiving TAT-GluR2 peptide (101.8±2.9%, *p*>0.05 versus control, *n* = 10) [Figure 5D,E]. Because GluR2 is located in VTA dopamine neurons as shown above, these data further support our hypothesis that HU210 induces *in vivo* transient synaptic depression in VTA dopamine neurons.

### Relation of Synaptic Depression and Cannabinoid Addictive Behavior

**Figure 6:**
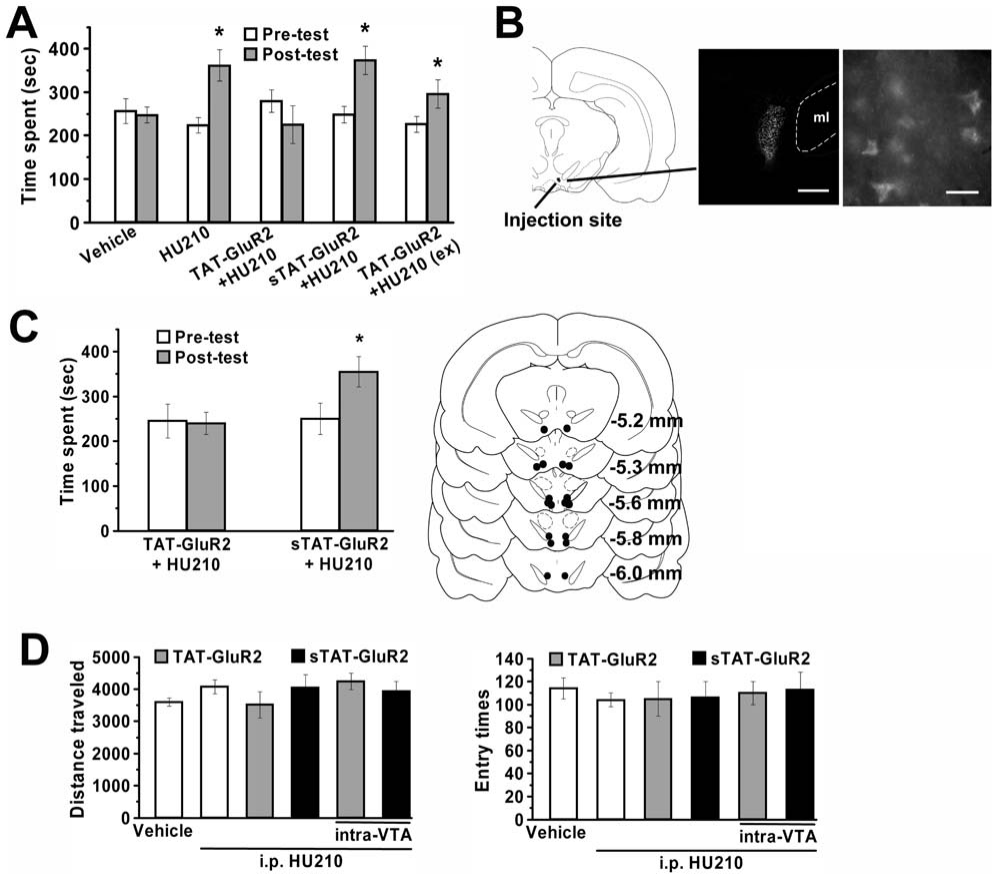
Blockade of HU210-elicited CPP by TAT-GluR2 peptide. (A) Systemic injection of TAT-GluR2 peptide, but not its scrambled version (sTAT-GluR2), blocked the acquisition, but not the expression (ex), of HU210-elicited CPP. One-way ANOVA: *F*_9,72_ = 3.132, *p*<0.01. * *p*<0.01 versus pre-test. (B) Diffusion of the fluorescent dansyl-TAT-GluR2 in the VTA after intra-VTA injection. Diagram (left) illustrates the diffusion area shown in the fluorescent image (middle). The right photograph shows peptide transduction in individual VTA neurons. ml, medial lemniscus. Scale bars: 400 µm (middle) or 25 µm (right). (C) Bilateral intra-VTA injection of TAT-GluR2 (*n* = 9), but not scrambled peptide (sTAT-GluR2; *n* = 9), blocked HU210-elicited CPP (left graph). * *p*<0.05 versus pre-test. The right histograms show reconstructions of histology sections illustrating VTA injection sites. (D) Effects of various treatments on locomotor activity. Animals receiving various treatments did not show significant difference in locomotor activity on the testing day, i.e. the total distance traveled in all 3 compartments (one-way ANOVA, *F*_5,43_ = 1.000, *p* = 0.430) and total entry times to each compartment (one-way ANOVA, *F*_5,43_ = 0.164, *p* = 0.975).

To explore whether VTA synaptic depression causatively contributes to cannabis addictive behavior, we examined the effects of synaptic depression blockade with TAT-GluR2 on HU210-induced conditioned place preference (CPP). Rats received TAT-GluR2 or its scrambled sequence (1.5 µmol/kg, i.p.) either at 2 h before each of four HU210 injections for conditioning or at 22 h after conditioning. Scrambled peptide exerted no significant effects on the acquisition of CPP (*p*<0.01 versus pre-test, *n* = 8), whereas TAT-GluR2 blocked the acquisition (*p*>0.05 versus pre-test, *n* = 7), but not the expression (*p*<0.05 versus pre-test, *n* = 8), of HU210-elicited CPP (Figure 6A). To determine whether systemically applied TAT-GluR2 peptide disrupted CPP acquisition by its action in the VTA, we conducted intra-VTA infusion of the peptide (15 pmol) 60 min before each HU210 injection during conditioning (Figure 6B). Consistent with our hypothesis, intra-VTA infusion of TAT-GluR2 peptide, but not scrambled peptide, blocked the acquisition of HU210-elicited CPP (TAT-GluR2: *p*>0.05 versus pre-test, *n* = 9; scrambled TAT-GluR2: *p*<0.05 versus pre-test, *n* = 9) (Figure 6C). Although cannabinoids can suppress motor function, the impact of HU210 on CPP appears independent of the motor function, because various treatments failed to affect locomotor activity on the testing day (Figure 6D).

## Discussion

A single injection of THC does not significantly affect synaptic plasticity at the local Glu-DA synapses [16]. In the present study, however we observed that chronic cannabinoid injection facilitated LFS-induced LTD, 24 h after the last cannabinoid exposure, at these synapses in a NMDAR- and AMPAR GluR2 endocytosis-dependent manner. Then, we hypothesized that cannabinoid exposure induced VTA synaptic depression in living animals, which lasts for less than 24 h. To support this hypothesis, we found that LFS failed to induce VTA LTD at 30 min after the fifth cannabinoid injection, indicating the occlusion of LFS-induced LTD *in vitro* by a preceding cannabinoid-induced LTD *in vivo*. Given that facilitated endocytosis of AMAPR GluR2 subunits is the last step of LTD expression [5], [19], [28], [29], our further observation of a significant endocytosis of GluR2 in the VTA at 30 min after cannabinoid injection also support our hypothesis. This hypothesis receives additional support by our findings suggesting that a blockade of GluR2 endocytosis prior to cannabinoid exposure with TAT-GluR2 prevented synaptic depression *in vivo*, resulting in a blockade of cannabinoid-facilitated *in vitro* LTD induction in the VTA.

AMPARs in inhibitory interneurons are devoid of GluR2 subunits in many brain regions such as the amygdala [31]–[34]. Here we provide the first evidence, to our knowledge, showing the localization of GluR2 in VTA dopamine neurons, instead of GABA neurons. Therefore, our findings of GluR2 endocytosis following cannabinoid exposure *in vivo* suggest that cannabinoid-induced *in vivo* synaptic depression occurs in VTA dopamine neurons, but not in GABA neurons. This idea is further supported by our whole cell recording experiments showing a significant decrease in AMPAR/NMDAR ratio of local VTA glutamatergic synaptic currents onto dopamine neurons 30 min following cannabinoid injection, because an increased AMPAR/NMDAR ratio of VTA glutamatergic synaptic currents onto dopamine neurons following cocaine exposure *in vivo* suggests cocaine-induced LTP *in vivo* in VTA dopamine neurons [12], [13].

Recent studies showed that mGluR-dependent LTD at VTA local Glu-DA synapses is distinct from that observed in the cerebellum and hippocampus [35], in which LTD results from AMPAR endocytosis [35], [36], while mGluR-dependent LTD in VTA dopamine neurons requires the exchange of GluR2-lacking AMPARs for those containing this subunit [16], [35]. In the present study we provide the first evidence, to our knowledge, suggesting that NMDAR-dependent LTD at VTA local Glu-DA synapses requires GluR2 endocytosis.

Endocannabinoids induce LTD via retrograde signalling from postsynaptic induction to presynaptic expression: postsynaptically released endocannabinoids act on presynaptic CB1R to induce presynaptic LTD via reduced release probability [19], [37], [38]. Thus, HU210 injection should induce a LTD that is expressed presynaptically via reduced glutamate release, as supported by a recent study showing that cannabinoids produce presynaptic inhibition of glutamatergic transmission onto VTA dopamine neurons [39]. It remains to be determined whether cannabinoid induces *in vivo* transient synaptic depression in VTA dopamine neurons through pre-, postsynaptic, or both mechanisms.

Wang's group shows that TAT-GluR2 peptide disrupted stress-induced impairment of spatial memory retrieval [26], amphetamine sensitization behavior [29], fear extinction [40], both GluR2 endocytosis and LTD induction, without significant effects on LTP induction, in the hippocampus [26] and nucleus accumbens [29]. Other three groups also show that the same peptide blocked fear conditioning-elicited LTD induction in the amygdala and fear extinction [41], [42], GluR2 endocytosis and decreased AMPAR/NMDAR ratio in the prefrontal cortex and cue-induced relapse to heroin seeking [43]. TAT-GluR2 produces no significant effects on basal synaptic transmission [29], which is in agreement with the specific role of the peptide in blocking regulated AMPAR endocytosis and hence the expression of LTD [28]. TAT-GluR2 produces no significant effects on motor activity, motivation to respond to amphetamine [29], or anxiogenic action [40]. TAT-conjugated peptides have been consistently shown to penetrate through the blood-brain-barrier and cell membrane so as to get into cells of all brain regions after a systemic administration [29], [44], [45]. Therefore, in the present study we utilized TAT-GluR2 for a selective disruption of LTD induction in both electrophysiological experiments and behavioral tests.

CPP produced by drugs of abuse results both from their direct rewarding effects and from their capacity in promoting learning to associate drug exposure with environmental cues [46]. The latter process, but not the innate rewarding response, requires long-term changes of synaptic strength in VTA dopamine circuitry [3], [46]. Thus, blockade of HU210-elicited CPP by TAT-GluR2 may represent its disruptive effects on learning to associate cannabinoid exposure with environmental cues. While numerous studies have reported long-term changes of synaptic strength at glutamatergic afferents onto VTA dopamine neurons [12]–[17], [35], [36], it is entirely unknown whether and how such changes in VTA synaptic plasticity causatively contributes to drug-associated addictive behavior. The present study provide the first direct evidence, to our knowledge, suggesting the causative role of AMPAR endocytosis-mediated transient synaptic depression at VTA local Glu-DA synapses in cannabinoid-associated addictive learning.

A single exposure to cocaine potentiated both VTA local Glu-DA synapses and PPN Glu-DA synapses through insertion of the higher conducting GluR2-lacking AMPAR into both synaptic pathways, which subsequently permitted expression of mGluR-dependent LTD through reinsertion of the lower conducting GluR2-containing AMPAR at these synapses [16]. However, a single exposure to THC induced GluR2-lacking AMPAR insertion into the PPN Glu-DA synapses without significant effects on the local Glu-DA synapses, thus permitting subsequent expression of mGluR-dependent LTD at the PPN Glu-DA synapses but not the local Glu-DA synapses [16]. This report also showed input-specificity for the baseline capacity for LTD at the PPN Glu-DA synapses and local Glu-DA synapses, indicating distinct roles played in each synaptic pathway. It remains to be determined whether and how cannabinoid-potentiated synaptic strength at VTA PPN Glu-DA synapses contribute to cannabinoid addictive behavior.

Similar to other drugs of abuse, cannabinoid is able to activate dopamine neuron [6], [7] and thus, a logical expectation is that all drugs of abuse would induce addiction through LTP induction in VTA dopamine neurons. In our pilot study HU210 (100 µg/kg, i.p.), which induced VTA LTD, failed to produce CPP in different strains of rats. Considering that rats resumed normal spatial learning at 24 h after the fifth daily HU210 injection (100 µg/kg, i.p.) [47], the findings in our pilot study may result from HU210-induced memory impairment effects during the first 4 days. We therefore modified protocol: rats were placed in their home cages after each of 4 daily priming HU210 injections; 24 h after the 4th priming injection, four conditioning pairs were conducted by placing rats in the non-preferred compartments for 30 min after HU210 injection. With this protocol we observed consistent CPP following HU210 injection. The CPP observed is unlikely produced by the anxiolytic effects of HU210 (100 µg/kg, i.p.) because this high dose of HU210 produce anxiogenic-like effects [48], although we cannot exclude the possibility that the CPP observed may be produced by the removal of some sort of conditioned place aversion. This protocol is different from other protocols for establishing rat CPP with other drugs of abuse, in which both 4-day prime training and 4-day conditioning in non-preferred environment are not required. Therefore, the development of cannabis-associated addictive learning, i.e. the learning to associate cannabis exposure with environmental cues, is different from the development of addictive behavior induced by other drugs of abuse.

Our data suggest that cannabinoid exposure induces *in vivo* transient synaptic depression in VTA dopamine neurons, but previous reports indicate that nicotine, cocaine, amphetamine, morphine and ethanol may induce LTP in these neurons [12]–[15]. If these drugs of abuse produce addictive behavior through VTA LTP, co-administration of cannabinoid and one of these drugs of abuse may neutralize their opposite effects on VTA synaptic plasticity and thereby block their individual addictive behavior during the development stage of drug addiction. This idea is supported by the findings that the cannabinoid WIN 55,212-2 inhibited cocaine addictive effects [49], [50]. However, recent studies indicate that cannabinoid may enhance or aggravate sensitization and relapse to other drugs of abuse because GluR2 endocytosis-dependent LTD may also be required for these processes. Thus, the same TAT-GluR2 peptide used in the present study to disrupt LTD induction prevented amphetamine-induced behavioral sensitization [29] and attenuated cue-induced relapse to heroin-seeking [43]; HU210 provoked relapse to cocaine seeking after prolonged periods of cocaine withdrawal [51].

### Conclusions

Our primary findings suggest that chronic cannabinoid exposure is able to alter long-term synaptic strength at VTA local Glu-DA synapses, although a recent study reveals no significant changes of synaptic plasticity at these synapses following a single exposure to cannabinoid [16]. While mGluR-dependent LTD in VTA dopamine circuitry requires the exchange of GluR2-lacking AMPARs for those containing this subunit [16], [35], we demonstrate here that NMDAR-dependent LTD in VTA dopamine circuitry requires GluR2 endocytosis. More importantly, while it is unknown whether alterations in synaptic plasticity in VTA dopamine circuitry causatively contribute to drug addictive behavior, our findings strongly suggest a pivotal role of LTD in VTA dopamine circuitry in the development of cannabinoid-associated addictive learning. In addition, the present study indicates a novel therapeutic strategy for treating cannabis addiction with the TAT-GluR2 peptide.

## Materials and Methods

### Animal Treatment

All procedures were performed in accordance with the guidelines established by the Canadian Council on Animal Care as approved by Animal Care Committee of the University of Ottawa Institute of Mental Health Research (IMHR ACC). Specifically, the IMHR ACC approved the present study (ACC-2007-001). Male Sprague-Dawley rats (16–92 days, Charles River) were employed, with young rats used for major part of field potential recording and all whole cell recording experiments and adult rats for some of field potential recording experiments and all molecular/biochemical, immunnohistochemical and behavioral studies. Because the amplitude of synaptic depression recorded from young rats is significantly higher than that from adult rats following chronic HU210 injection, as illustrated by Figures 1 (young rats) and 2 (adult rats), data comparison was done only between young or adult rats and the same control groups were used in both young and adult rats. Rats received no treatment (naïve rats) or once daily injections for 5 days of HU210 (100 µg/kg, i.p.), THC (5 mg/kg, i.p.), or vehicle. In some cases, AM281 (3 mg/kg, i.p.) and TAT-GluR2 peptide (1.5 µmol/kg, i.p.) or scrambled TAT-GluR2 peptide was injected 20 min (AM281) or 2 h before HU210 injection. Intra-VTA cannulation was conducted under isoflurance anesthesia. Rats received bilateral cannulae (26 Ga) implantation into the VTA (−5.8 AP, +0.75 ML, −7.5 DV), and then cannula were secured to the skull. One week later, adenoviral vectors (10^10^ plaque-forming units/µl/side, a single infusion 1 day before the first daily HU210 injection) or a dansyl tagged TAT-GluR2 peptide (15 pmol in 0.6 µl) was injected into the VTA. The diffusion regions of adenoviral vectors-containing green fluorescent protein (GFP) and Dansyl-TAT-GluR2 were examined after perfusion rats transcardially and cutting brain sections as described [18], [52].

### Electrophysiology

The VTA fEPSPs were recorded as described [17]. Briefly, rats anaesthetized with chloral hydrate were decapitated 30 min or 24 h after the last injection of HU210 or vehicle. A block of tissue containing midbrain was rapidly removed into ice-cold low-calcium artificial cerebrospinal fluid (ACSF) containing (in mM): 126 NaCl, 2.5 KCl, 1.2 NaH_2_PO_4_, 1.2 MgSO_4_, 0.625 CaCl_2_, 21.4 NaHCO_3_, 11.1 glucose, with 0.4 ascorbic acid added. Horizontal slices (320 µm) were cut with a vibrotome and stored for at least 1 h at 35°C in the standard ACSF (i.e., containing 2.4 mM CaCl_2_) with 0.4 mM ascorbic acid added. Slices were transferred into recording chamber (30–32°C) and continuously perfused with the standard ACSF at 1.5–2.0 ml/min. Extracellular and whole cell recordings from the VTA were performed with a Multiclamp 700B amplifier (Axon Instruments). Both fEPSP and EPSC were evoked at 0.05 Hz with a concentric bipolar electrode (FHC) placed 100–300 µm (for extracellular recording) or 50–100 µm (for intracellular recording) rostral to the recording site. Stimulus pulse intensities were typically 0.3–1.5 mA with duration of 300 µs for fEPSP or 100 µs for EPSC. For extracellular recording, a glass microelectrode was filled with 2 M NaCl. For whole-cell recordings with perforated patch, individual cells in the VTA were visualized using infrared-differential interference contract. For whole cell LTD recording, patch pipettes (4–7 MΩ) were filled with a solution containing (in mM): 140 potassium gluconate, 5 KCl, 10 HEPES, 0.2 EGTA, 2 MgCl_2_, 4 MgATP, 0.3 Na_2_GTP and 10 Na_2_-phosphocreatine, with pH 7.2 (adjusted by KOH. 1 mg/ml). Perforated-patch was performed by adding DMSO-dissolved amphotericin B into the internal solution, and neurons were voltage-clamped at −70 mV. Dopamine and GABA cells were identified by the presence and absence of a large (>50 pA) *I*_h_ current, respectively, in response to a hyperpolarizing pulse to −110 mV upon break in [9]. Because the presence of a large *I*_h_ current does not exclusively belong to dopamine neurons [27], putative dopamine neurons in our study may contain some non-dopamine neurons. LTP was induced with HFS at 100 Hz for 1 sec and this train was repeated twice, 20 s apart. LTD was elicited with LFS at 1 Hz for 15 min. Levels of LTP or LTD are reported as a comparison of average EPSC or fEPSP amplitudes for a 10 min time period immediately before LTP or LTD induction with those during the 10 min period from 40 to 50 min after HFS or LFS [17], [53]. To measure the AMPAR/NMDAR ratio, the intracellular solution contained (in mM): 120 CsCH_3_SO_3_, 20 HEPES, 0.4 EGTA, 2.8 NaCl, 5 TEA-Cl, 2 MgCl_2_, 2.5 MgATP, 0.3 GTP (pH 7.2 with CsOH); dopamine neuron was voltage-clamped at +40 mV. After a stable baseline recording of total EPSCs, the NMDAR antagonist DL-2-Amino-5-phosphonopentanoic acid (AP-5, 50 µM) was applied in the bath for 5–10 min to isolate fast AMPAR-mediated EPSCs, and digital subtraction of AMPAR-EPSCs from the total EPSCs from the same neuron yielded NMDAR-EPSCs.

### Adenovirus Preparation and *in vivo* Gene Silencing

The oligonucleotides encoding scramble or rat CB1-targeted shRNAs were synthesized, annealed and ligated into the GFP-expressing adenoviral shuttle vector pAdshRNA/H1 [54]. The targeting sequences of shRNAs are as follows: CB1-1, 5′-GATGAACAAGCTTATCAAG-3′ and CB1-2, 5′-CTGCAAGAAGCTGCA ATCT-3′; scramble, 5′-GAGAGTACTACGATCATAA-3′. Recombinant adenoviruses were prepared using the pAdEasy system [55]. After intra-VTA microinfusion of adenoviral vectors, the VTA was dissected for the quantification of CB1R protein using immunoblotting procedures described below. Transduction of VTA cells by recombinant adenoviruses was determined by GFP reporter coexpressed by the vector.

### Immunofluorescent Staining

Using protocol described [52], rats were perfused with 4% paraformaldehyde and brains were cut into frontal sections (40-µm thick), which were then incubated in a cocktail solution containing mouse anti-GluR2 antibody (1∶100; Chemicon) and sheep anti-TH antibody (1∶100; Chemicon) or rabbit anti-GAD65/67 antibody (1∶100; Chemicon) for 3 days. After washing, the sections were incubated in another cocktail solution containing Alexa Fluor 568-conjugated goat anti-rabbit (1∶500, Molecular Probes) and Alexa Fluor 488-conjugated goat anti-mouse (1∶500, Molecular Probes) or donkey anti-sheep (preabsorbed with goat IgG) secondary antibodies (1∶1000, Molecular Probes) at room temperature for 4 h. For GFP single imuunohistochemical labeling, GFP was visualized with mouse anti-GFP antibody (1∶2000, Sigma). Sections were examined with 10× or 60× lens under confocal laser scanning microscopes (Zeiss, LSM510 META).

### Surface AMPAR Measurement

Biotinylation experiments were performed with modifications of the recent protocols [56], [57]. Briefly, after incubation in ACSF containing 1 mg/ml of Sulfo-NHS-SS-Biotin (Pierce) for 30 min, midbrain slices were rinsed 3 times and then homogenized in 500 µl of lysis buffer (1% triton-X100, 0.1% SDS, 50 mM Tris pH 7.4, 150 mM NaCl, 2 mM EDTA, 0.5 mM AEBSF, 50 µM Antipain HCl, 25 µM leupeptine and 0.5 µg/ml pepstatin), followed by centrifugation at 14,000×g for 15 min at 4°C. While 25 µl of biotinylated proteins were used as the “total” fraction, 50 µl of streptavidin beads (Sigma) were added to 100 µg of biotinylated VTA proteins and incubated overnight at 4°C. After washing, biotinylated proteins were eluted off the beads using 40 µl of 2× SDS-PAGE loading buffer. Then, immunoblotting was conducted as described below.

### Immunoblotting

Immunoblotting was conducted as described [52]. Briefly, membranes were probed with primary antibodies to GluR1 (1∶250, Upstate), GluR2 (1∶500, Chemicon), GluR3 (1∶200, Chemicon) or CB1R (1∶500, Santa Cruz Biotechnology) overnight at 4°C. Immunoreactive bands were visualized using enhanced chemiluminescence and captured by a CCD digital camera. Bands were analyzed by densitometry (Alpha Innotech). Receptor ratio for AMPAR subunits was determined by dividing the surface intensity by the total intensity.

### Behavioral Tests

The place preference system (TSE Systems, Inc., Midland, USA) consisted of 3 distinct chambers with non-transparent walls. CPP was conducted with modifications of our recent protocol [52]. Briefly, CPP took place over preconditioning, priming (HU210 only), conditioning and postconditioning phases. During preconditioning, which was conducted by allowing rats to have free access to 3 chambers for 15 min per day for 1 or 2 days, the environment was adjusted to allow rats to stay in one choice chamber for longer time (i.e. the preferred chamber) than the other choice chamber (i.e. the none-preferred chamber). During priming phase rats were placed in their home cages for 1 day immediately after each daily injection of HU210 (100 µg/kg, i.p.) for 4 days. For the conditioning, which was conducted 24 h after the last HU210 injection for cannabinoid priming, four pairings with drug and four pairings with vehicle were conducted once daily for 8 days. Ten min after an i.p. injection of HU210 (100 µg/kg) or vehicle (HU210∶DMSO∶TWEEN 80∶saline = 1∶1∶8), rats were placed in the none-preferred conditioning compartment for 30 min, with the gateway closed. On alternative days, all groups of rats were given vehicle injection and then placed in the preferred compartment for 30 min, with the gateway closed. For study the effects of TAT-GluR2 one the acquisition of CPP, rats received i.p. or bilateral intra-VTA injections of TAT-GluR2 peptide or scrambled TAT-GluR2 peptide 1 h (15 pmol/0.6 µl/side, intra-VTA) or 2 h (1.5 µmol/kg, i.p.) before HU210 injection (100 µg/kg, i.p.). All intra-VTA injections were administered in a volume of 0.6 µl/side. On alternative days, rats received double injections of appropriate vehicle (saline for the peptide). The postconditioning was conducted exactly as in the preconditioning, i.e. free access to each compartment for 15 min, during which the time spent by each rat in the none-preferred compartment was recorded. The time spent by each rat in the none-preferred compartment during the preconditioning and postconditioning was compared, with higher and lower values in post-test indicating CPP and conditioned place aversion, respectively. For study the effects of TAT-GluR2 on the expression of CPP, rats received an i.p. injection of TAT-GluR2 peptide or scrambled TAT-GluR2 peptide 2 h (1.5 µmol/kg, i.p.) before the postconditioning. The total distance traveled in all 3 compartments and total entry times to each compartment during postconditioning were recorded for measuring locomotor activity. After postconditioning, rats receiving intra-VTA injections were perfused transcardially and brain sections were stained with cresyl violet for verification of cannula placement.

### Data Analysis

Results were reported as mean ± SE. Statistical analysis of the data was performed using a student *t* test, one-way ANOVA or one-way ANOVA for repeated measures, followed by Fisher's LSD *post hoc* test. Statistical significance was set at *p*<0.05.
